# Case Report: Positive Pressure Therapy Combined With Endolymphatic sac Surgery in a Patient With Ménière's Disease

**DOI:** 10.3389/fsurg.2021.606100

**Published:** 2021-03-25

**Authors:** Munehisa Fukushima, Shiro Akahani, Hidenori Inohara, Noriaki Takeda

**Affiliations:** ^1^Department of Otolaryngology—Head and Neck Surgery, Kansai Rosai Hospital, Amagasaki, Japan; ^2^Department of Otolaryngology—Head and Neck Surgery, Graduate School of Medicine, Osaka University, Osaka, Japan; ^3^Department of Otolaryngology, University of Tokushima School of Medicine, Tokushima, Japan

**Keywords:** positive pressure therapy, Ménière's disease, endolymphatic hydrops, endolymphatic sac surgery, magnetic resonance imaging

## Abstract

Positive pressure therapy (PPT) is applied for medically-intractable vertigo in Ménière's disease (MD); however, it remains unknown whether PPT affects *in vivo* endolymphatic hydrops (EH). In this case report, we describe a 5-year course of MD in a patient in which EH was repeatedly observed. As the patient experienced recurrent vertigo attacks after endolymphatic sac surgery, he began to use the PPT device additionally and vertiginous episodes decreased in accordance with a decrease in the EH volume. The mechanism of PPT is suggested that the pressure increase in the middle ear inhibits EH development. PPT, if added after surgery, might be more effective to reduce EH volume compared with surgery alone. A larger study group size is required to test these preliminary data concerning EH changes.

## Introduction

Ménière's disease (MD) is a common inner ear disease that is characterized by episodic vertigo, fluctuating sensorineural hearing loss, and tinnitus. Twenty percent of patients with MD are refractory to medical therapy (1) and suffer frequent vertigo attacks with progressive profound hearing loss (2). For medically-intractable MD patients, available options other than function-ablative procedures are positive pressure therapy (PPT) or endolymphatic sac surgery (3), although review articles concluded insufficient evidence to support the benefit of both PPT and surgery (4, 5). MD is pathologically defined as idiopathic endolymphatic hydrops (EH) in the inner ear (6, 7), and reducing EH is an hypothesized pathway in these two therapies; however, this has not been directly demonstrated for PPT.

EH is currently easily visualized using 3-Tesla magnetic resonance imaging (MRI) after intravenous administration of gadolinium (Gd) (8). Using this imaging method, we routinely characterize EH enlargement in patients with MD and measure EH volume semi-quantitatively (9). In this short preliminary report, we describe a 5-year course of MD in a patient in which EH volume was repeatedly measured, and demonstrated *in vivo* EH reduction using a PPT device. The significance of these findings is discussed with specific reference to known EH pathophysiology.

Informed consent was obtained from the described patient.

## Case Report

**Figure 1:**
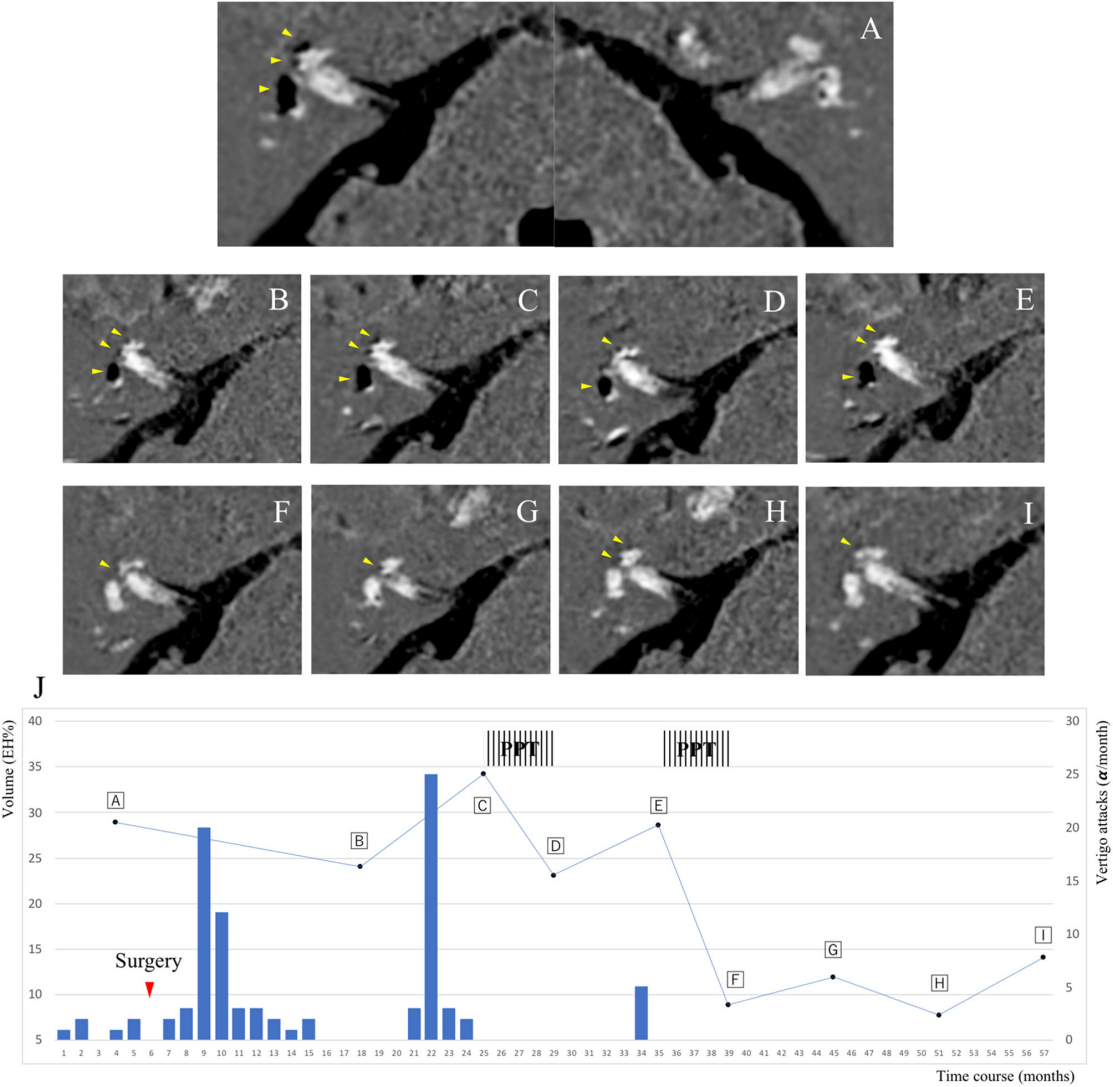
Course of endolymphatic hydrops (EH) and progression of vertigo attacks in a 68-year-old man. The patient's course was semi-quantitatively assessed by gadolinium-enhanced inner ear magnetic resonance images, which are shown in **(A–I)** (axial T2-weighted fluid-attenuated inversion recovery). The y axis on the left indicates the volume ratio of EH to the total inner ear volume in the right ear. **(J)** is a progress chart of the patient's vertigo attacks as frequency per month on the y axis (right-hand side). **(A)** Significant EH in the right vestibule and cochlea. No EH in the left vestibule and cochlea. May 2015. **(B)** Mild EH in the right vestibule and cochlea. July 2016. **(C)** Significant EH in the right vestibule and mild EH in the right cochlea. February 2017. **(D)** Mild EH in the right vestibule and cochlea. June 2017. **(E)** Significant EH in the right vestibule and mild EH in the right cochlea. December 2017. **(F)** No EH in the right vestibule and mild EH in the right cochlea. April 2018. **(G)** October 2018. **(H)** April 2019. **(I)** November 2019. No EH in the right vestibule and mild EH in the right cochlea. The yellow arrowheads indicate EH, and the black areas represent EH in the labyrinth. **(J)** The red inverted triangle in the chart indicates the day of surgery. The vertical stripes indicate the durations of the positive pressure therapy.

A 68-year-old man, a piano instructor, complained of repeated vertigo for a few hours with nausea once per month for 15 years despite taking medications, namely diuretics and difenidol. He suffered from persistent tinnitus in the right ear, and pure-tone audiometry indicated sensorineural hearing loss of 48.8/37.5 dB involving the whole frequency spectrum [right/left ears, four-tone average according to the AAO-HNS criteria (10)]. He underwent Gd-enhanced MRI of the inner ear and neuro-otological testing. The first MRI scan revealed significant EH in the right vestibule and cochlea (Figure 1A); the volume ratio of EH relative to total inner ear volume (EH%) was 28.8%. The bithermal water-irrigation caloric test was used to measure the maximum slow phase velocity, and results showed no response in the right ear. We diagnosed right definite MD (stage 3) (10), educated the patient regarding diet and lifestyle modifications, and prescribed betahistine and diuretics. After 4 months of the additional treatments, the frequency of the vertigo attacks remained constant, and hearing in his right ear worsened to 56.3 dB. We performed endolymphatic sac drainage with steroid instillation (11) on July 2015. We followed the patient to evaluate vertigo and hearing at least once per month and requested that he record the date, severity, and duration of vertigo attacks in a self-check diary (12); the course of the vertigo from the month prior to the first examination is shown in Figure 1J. In May 2016, he reported no vertigo, and the second MRI revealed decreased EH (Figure 1B, EH% = 24). However, he suffered frequent vertigo attacks beginning in October 2016, hearing in his right ear worsened to 66.3 dB, and the third MRI revealed increased EH (Figure 1C, EH% = 34.2). He began to use the PPT device (EFET01, Daiichi Medical Co., Ltd., Japan) for the first time at home three times daily from February 2017 to June 2017. Vertiginous episodes resolved, hearing in his right ear improved to 55 dB, and the fourth MRI revealed decreased EH (Figure 1D, EH% = 23). However, vertiginous episodes recurred in November 2017, hearing in his right ear worsened to 65 dB, and the fifth MRI revealed increased EH (Figure 1E, EH% = 28.5). The second series of using the PPT device at home was performed from December 2017 to April 2018. Vertiginous episodes decreased, hearing in his right ear improved to 46.3 dB, and the sixth MRI revealed decreased EH equal to normal volume (Figure 1F, EH% = 8.8). For 2 years beginning in December 2017, he reported no vertigo, and the subsequent MRI revealed overall low EH values (Figures 1G–I: EH% = 11.8, 7.7, and 14, respectively). The latest hearing level was 47.5/42.5 dB, and he showed no caloric response in his right ear. All nine MRI scans detected no EH in the left inner ear throughout the 5-year observational course.

## Discussion

**Figure 2:**
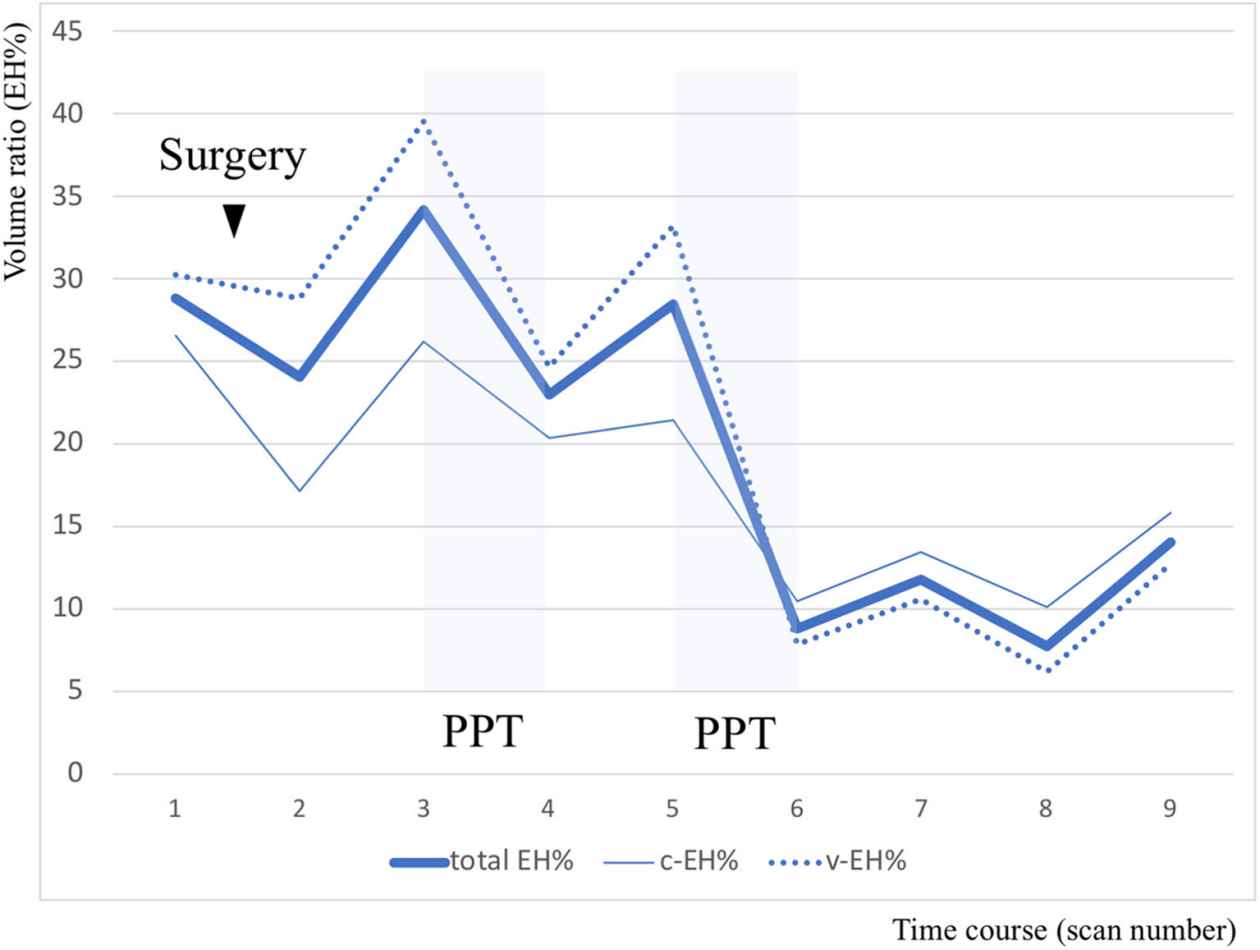
Volume analysis of endolymphatic hydrops (EH) divided into cochlea and vestibule regions. The y axis indicates the volume ratio of EH to the involved part of the inner ear volume in the right ear. In this chart, the bold line indicates the volume ratio of EH relative to total inner ear volume (total EH%), the fine line indicates the volume ratio of EH relative to cochlear volume (c-EH%), and the dotted line indicates the volume ratio of EH relative to vestibular volume (v-EH%).

PPT and endolymphatic sac surgery is recommended as a second-line therapy for intractable MD when various medications fail (3). Several reports using MRI described EH volume decrease after sac surgery (13–15); however, to the best of our knowledge, no previous reports have demonstrated the effects of PPT for *in vivo* EH volume change in MD patients. In this patient, the frequency of the vertigo attacks was fully correlated with increases and decreases in EH volume (Figure 1J). There is a close association between the vector of the EH volume and MD symptoms, and the decreased EH% values were greater than twice the values after adding PPT (from 34.2 to 23 and from 28.5 to 8.8, respectively) than with surgery only (from 28.8 to 24). As shown in Figure 2 and Table 1, EH volume in the right ear of this case showed almost parallel changes, both when evaluated by total volume and by each region. The frequency of the monthly vertigo attacks increased from 1 to 10 after surgery, but decreased from 10 to 0 after adding PPT; the decrease in vestibular EH might have reflected vestibular symptoms in this case. PPT, which was added 18 months after surgery, might be more effective to reduce the frequency of vertigo and the EH volume compared with surgery alone.

**Table 1:** Sequential values of the volume ratio of endolymphatic hydrops (EH) in the right ear according to region.

	**1**	**2**	**3**	**4**	**5**	**6**	**7**	**8**	**9**
total EH%	28.8	24	34.2	23	28.5	8.8	11.8	7.7	14
c-EH%	26.5	17.1	26.2	20.4	21.4	10.4	13.4	10.1	15.8
v-EH%	30.3	28.8	39.5	24.7	33.2	7.8	10.6	6.2	12.7

*Total EH%, volume ratio of EH to total inner ear volume; c-EH%, volume ratio of EH to cochlear volume; v-EH%, volume ratio of EH to vestibular volume*.

A meta-analysis of PPT reported a reduction in vestibular symptoms in patients with MD (16, 17), but the clinical efficacy of PPT for MD remains controversial (4). Animal studies showed that positive middle ear pressure instantly transferred to inner ear pressure (18), and electrocochleography recordings demonstrated that the summating potential significantly decreased in the PPT group (19). The suggested mechanism of PPT is that the pressure increase in the middle ear improves endolymphatic drainage and inhibits EH development (20), and this hypothesis was proven for the first time, in this study. The PPT device used in our study, unlike the Meniett device, provides intermittent positive pressure without ventilation tube insertion, and vertigo control for MD patients was demonstrated to be as effective as with the Meniett device (21). With these considerations, this remarkable case suggests that local pressure pulse application without oxygenation can affect labyrinthine physiology and induce *in vivo* EH reduction. Regarding our patient's hearing level in the affected ear, the difference between the first and the latest audiometry of 1.3 decibels was considered no change. Although the hearing level in the affected ear is reported to correlate with EH volume (22), the decrease in cochlear EH might not have improved the hearing level in this case. In MD, hearing levels worsen, and EH volume develops over time (23). The EH-reducing effect of a PPT device might have stopped the hearing deterioration, in our patient. Additionally, image data before or after PPT are useful to accurately determine the results of treatment.

In summary, we successfully treated a patient with intractable MD using a PPT device combined with endolymphatic sac surgery. Positive pressure could remedy vertiginous symptoms of MD through EH volume reduction. As this is a single case, and the data are preliminary, a larger study group size is required to evaluate the effect of PPT for *in vivo* EH. We plan to address this limitation in a future study.

## Data Availability Statement

The original contributions presented in the study are included in the article/supplementary material, further inquiries can be directed to the corresponding author/s.

## Ethics Statement

Ethical review and approval was not required for the study on human participants in accordance with the local legislation and institutional requirements. The patients/participants provided their written informed consent to participate in this study. Written informed consent was obtained from the individual(s) for the publication of any potentially identifiable images or data included in this article.

## Author Contributions

MF initiated and performed the surgery. All authors were involved in the writing, reviewing, and editing of the manuscript.

## Conflict of Interest

The authors declare that the research was conducted in the absence of any commercial or financial relationships that could be construed as a potential conflict of interest.
